# Supplementary material for: The Histone H3K27 Demethylase REF6 Is a Positive Regulator of Light-Initiated Seed Germination in Arabidopsis
Source: Cells. 2023 Jan 12;12(2):295. doi: 10.3390/cells12020295 (PMC9856397; doi:10.3390/cells12020295)
Supplement: Supplementary file 1 [file cells-12-00295-s001.zip › Supplemental Figure S1.pptx]

## Slide 1
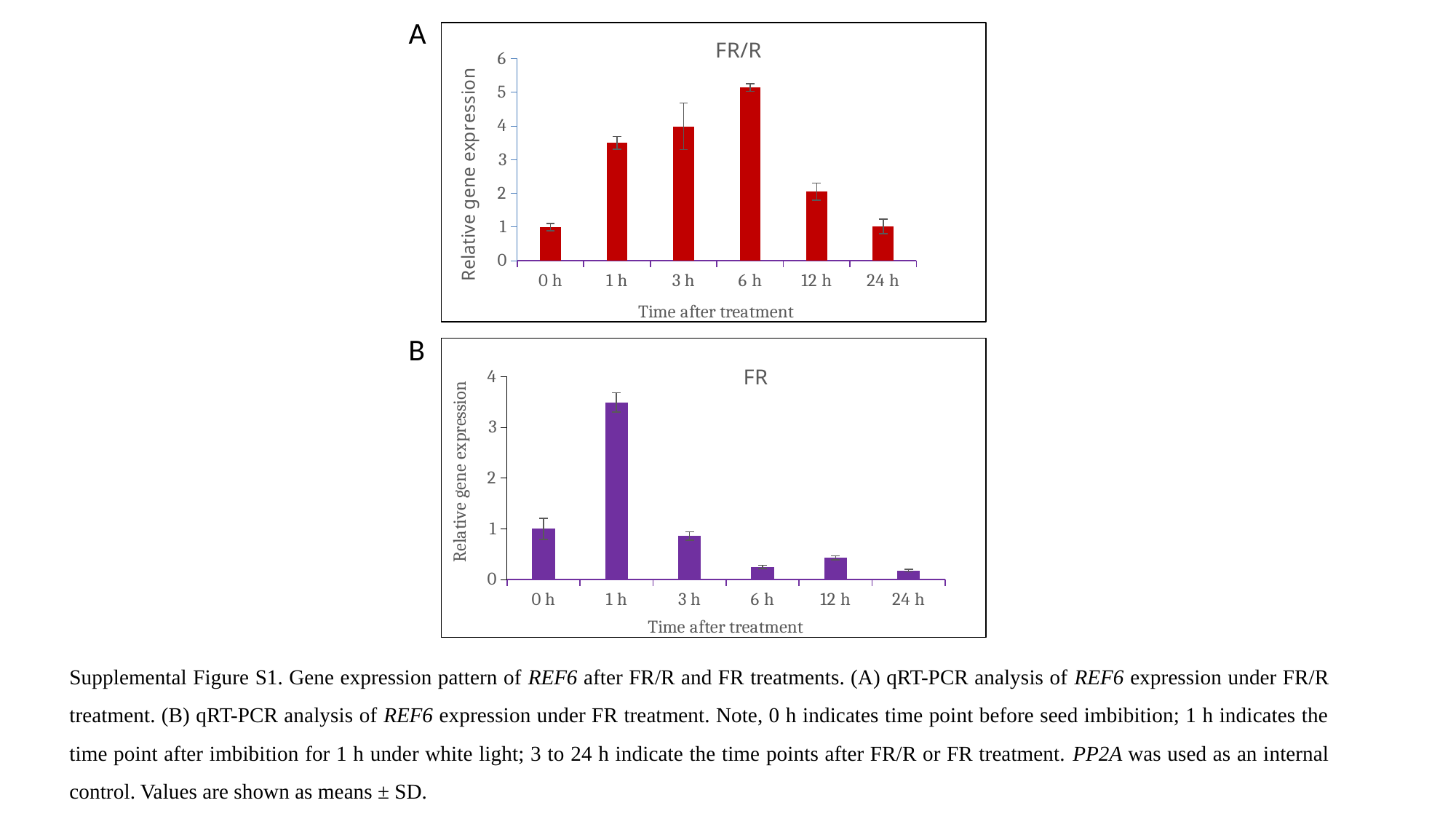

A
### Chart: FR/R
| Category | |
|---|---|
| 0 h | 1.0 |
| 1 h | 3.4962231251763685 |
| 3 h | 3.9906427502677495 |
| 6 h | 5.138234778086318 |
| 12 h | 2.0503541110939354 |
| 24 h | 1.0193022066304764 |B
### Chart: FR
| Category | |
|---|---|
| 0 h | 1.0 |
| 1 h | 3.4962231251763685 |
| 3 h | 0.8575701948003075 |
| 6 h | 0.24815087429175997 |
| 12 h | 0.4288846918229451 |
| 24 h | 0.17643821562052983 |Supplemental Figure S1. Gene expression pattern of REF6 after FR/R and FR treatments. (A) qRT-PCR analysis of REF6 expression under FR/R treatment. (B) qRT-PCR analysis of REF6 expression under FR treatment. Note, 0 h indicates time point before seed imbibition; 1 h indicates the time point after imbibition for 1 h under white light; 3 to 24 h indicate the time points after FR/R or FR treatment. PP2A was used as an internal control. Values are shown as means ± SD.
