# Supplementary material for: The Histone H3K27 Demethylase REF6 Is a Positive Regulator of Light-Initiated Seed Germination in Arabidopsis
Source: Cells. 2023 Jan 12;12(2):295. doi: 10.3390/cells12020295 (PMC9856397; doi:10.3390/cells12020295)
Supplement: Supplementary file 1 [file cells-12-00295-s001.zip › Supplemental Figure S2.pptx]

## Slide 1
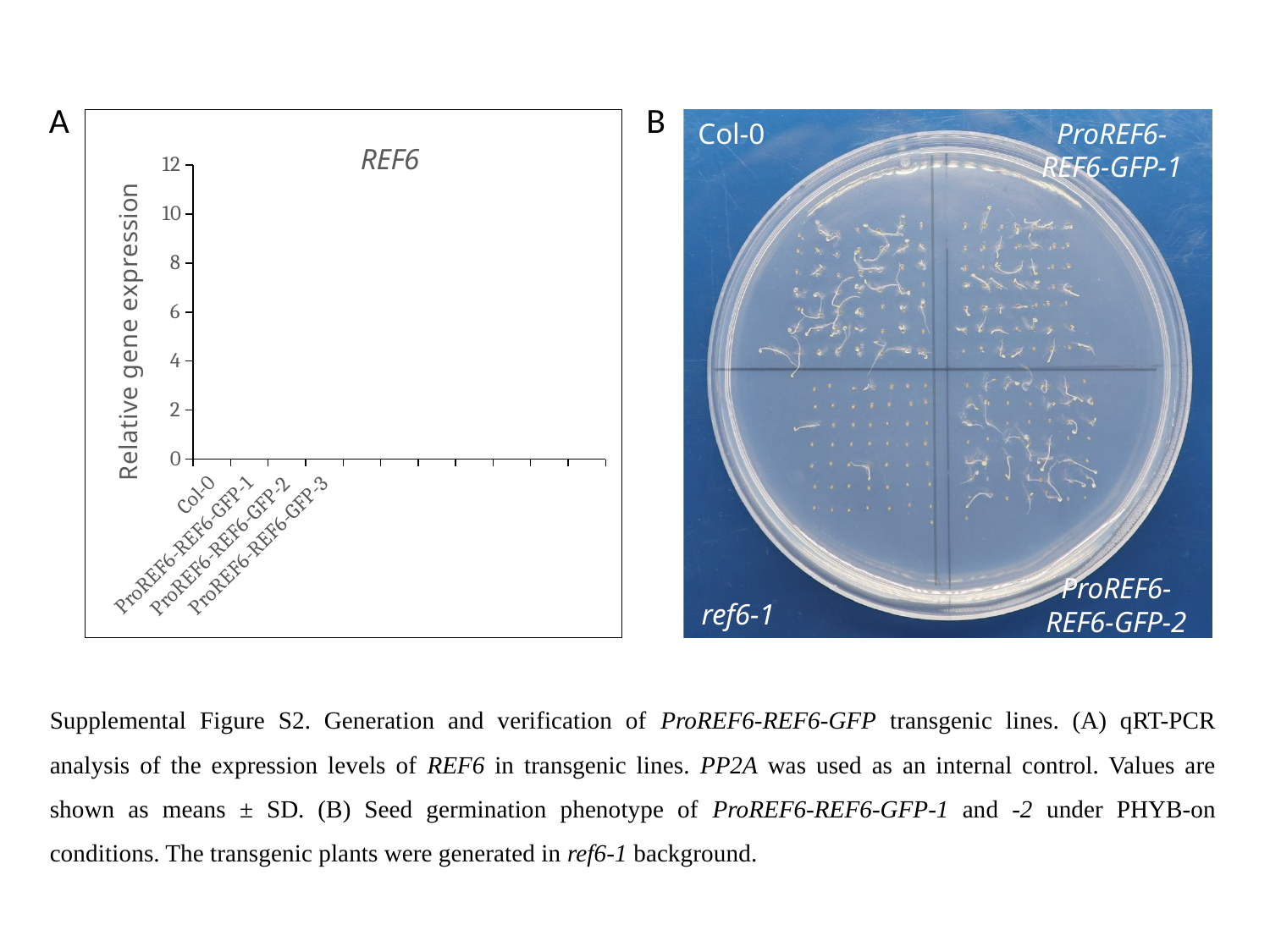

A
B
### Chart: REF6
| Category | |
|---|---|
| Col-0 | 1.0 |
| ProREF6-REF6-GFP-1 | 1.6181287468525052 |
| ProREF6-REF6-GFP-2 | 1.217284686467933 |
| ProREF6-REF6-GFP-3 | 1.796427759004428 |
Col-0
ProREF6-REF6-GFP-1
ProREF6-REF6-GFP-2
ref6-1
Supplemental Figure S2. Generation and verification of ProREF6-REF6-GFP transgenic lines. (A) qRT-PCR analysis of the expression levels of REF6 in transgenic lines. PP2A was used as an internal control. Values are shown as means ± SD. (B) Seed germination phenotype of ProREF6-REF6-GFP-1 and -2 under PHYB-on conditions. The transgenic plants were generated in ref6-1 background.
