# Supplementary material for: The Histone H3K27 Demethylase REF6 Is a Positive Regulator of Light-Initiated Seed Germination in Arabidopsis
Source: Cells. 2023 Jan 12;12(2):295. doi: 10.3390/cells12020295 (PMC9856397; doi:10.3390/cells12020295)
Supplement: Supplementary file 1 [file cells-12-00295-s001.zip › Supplemental Figure S3.pptx]

## Slide 1
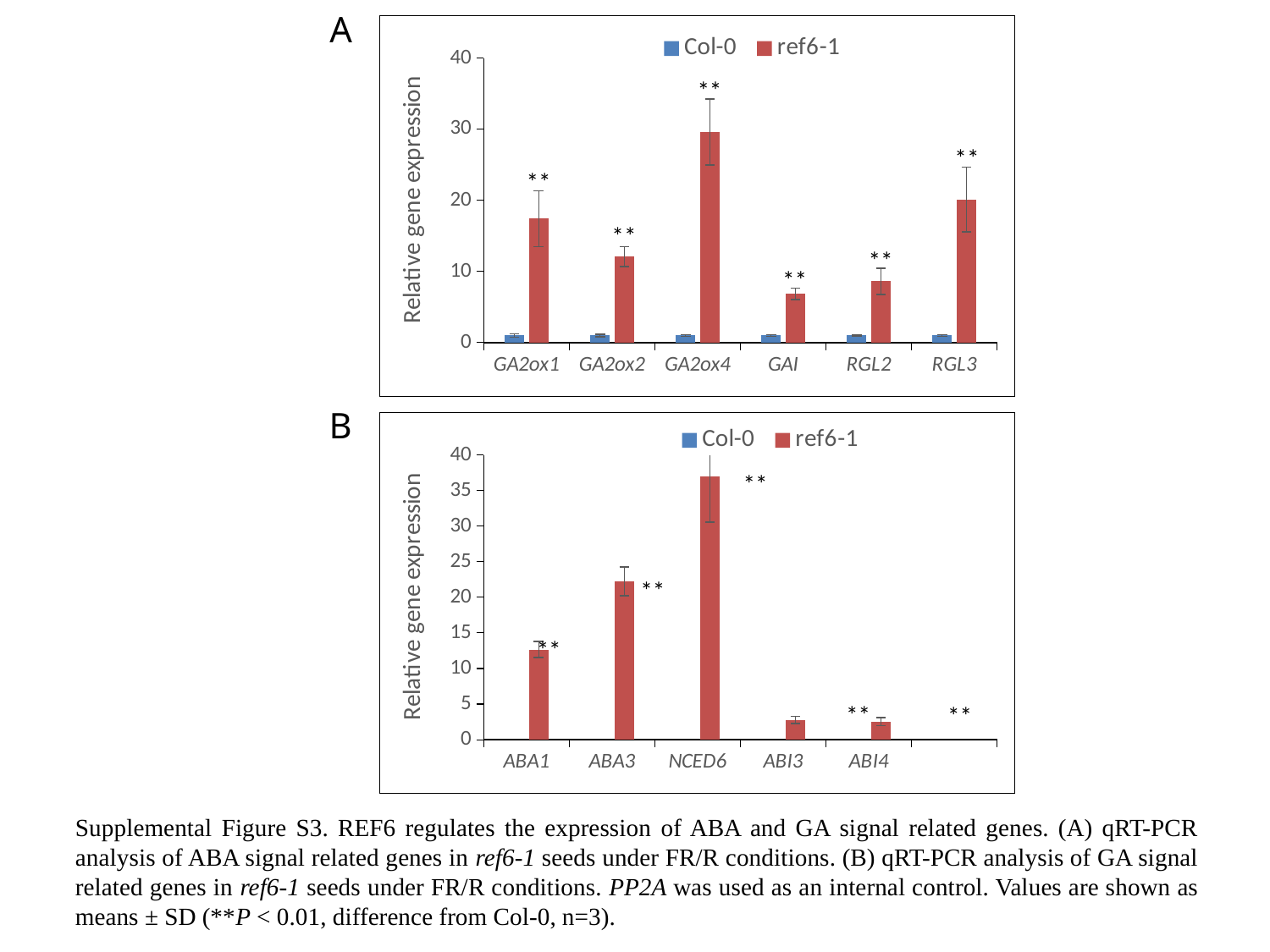

A
### Chart
| Category | Col-0 | ref6-1 |
|---|---|---|
| GA2ox1 | 1.0 | 17.436334346266513 |
| GA2ox2 | 1.0 | 12.09000703804447 |
| GA2ox4 | 1.0 | 29.59969283803269 |
| GAI | 1.0 | 6.86730551551959 |
| RGL2 | 1.0 | 8.622428230795085 |
| RGL3 | 1.0 | 20.13482698001013 |**
**
**
**
**
**
B
### Chart
| Category | Col-0 | ref6-1 |
|---|---|---|
| ABA1 | 1.0 | 12.665912818315174 |
| ABA3 | 1.0 | 22.257480434484524 |
| NCED6 | 1.0 | 37.05134256109689 |
| ABI3 | 1.0 | 2.754361809314089 |
| ABI4 | 1.0 | 2.5214344643353557 |**
**
**
**
**
Supplemental Figure S3. REF6 regulates the expression of ABA and GA signal related genes. (A) qRT-PCR analysis of ABA signal related genes in ref6-1 seeds under FR/R conditions. (B) qRT-PCR analysis of GA signal related genes in ref6-1 seeds under FR/R conditions. PP2A was used as an internal control. Values are shown as means ± SD (**P < 0.01, difference from Col-0, n=3).
